# The impact of physical exercise on the fatigue symptoms in patients with multiple sclerosis: a systematic review and meta-analysis

**DOI:** 10.1186/s12883-020-01654-y

**Published:** 2020-03-13

**Authors:** Nazanin Razazian, Mohsen Kazeminia, Hossein Moayedi, Alireza Daneshkhah, Shamarina Shohaimi, Masoud Mohammadi, Rostam Jalali, Nader Salari

**Affiliations:** 1 Department of Neurology, School of Medicine, Kermanshah University of Medical Sciences, Kermanshah, Iran; 2 Department of Nursing, School of Nursing and Midwifery, Kermanshah University of Medical Sciences, Kermanshah, Iran; 3 Institute of Research and Development, Ton Duc Thang University, Da Nang, 550000 Viet Nam; 4 School of Computing, Electronics and Maths, Coventry University, London, UK; 5 Department of Biology, Faculty of Science, University Putra Malaysia, Serdang, Selangor Malaysia; 6 Department of Biostatistics, School of Health, Kermanshah University of Medical Sciences, Kermanshah, Iran

**Keywords:** Physical exercise, Fatigue, Multiple sclerosis

## Abstract

**Background:**

Despite many benefits of the physical activity on physical and mental health of patients with Multiple Sclerosis (MS), the activity level in these patients is still very limited, and they continue to suffer from impairment in functioning ability. The main aim of this study is thus to closely examine exercise’s effect on fatigue of patients with MS worldwide, with particular interest on Iran based on a comprehensive systematic review and meta-analysis.

**Methods:**

The studies used in this systematic review were selected from the articles published from 1996 to 2019, in national and international databases including SID, Magiran, Iranmedex, Irandoc, Google Scholar, Cochrane, Embase, ScienceDirect, Scopus, PubMed and Web of Science (ISI). These databases were thoroughly searched, and the relevant ones were selected based on some plausible keywords to the aim of this study. Heterogeneity index between studies was determined using Cochran’s test and I^2^. Due to heterogeneity in studies, the random effects model was used to estimate standardized mean difference.

**Results:**

From the systematic review, a meta-analysis was performed on 31 articles which were fulfilled the inclusion criteria. The sample including of 714 subjects was selected from the intervention group, and almost the same sample size of 720 individuals were selected in the control group. Based on the results derived from this meta-analysis, the standardized mean difference between the intervention group before and after the intervention was respectively estimated to be 23.8 ± 6.2 and 16.9 ± 3.2, which indicates that the physical exercise reduces fatigue in patients with MS.

**Conclusion:**

The results of this study extracted from a detailed meta-analysis reveal and confirm that physical exercise significantly reduces fatigue in patients with MS. As a results, a regular exercise program is strongly recommended to be part of a rehabilitation program for these patients.

## Background

Multiple Sclerosis, a chronic disease which has a high prevalence in the world and also Iran, is a worldwide problem which requires considerable time and enormous financial resource to achieve the rehabilitation targets [1, 2]. Based on the scientific epidemiological studies, nearly 52.9 in one hundred thousand (100 k) people in Iran are affected with this disease, such that the prevalence of this disease in Tehran has reached 50 in 100 k people. This rate is very similar to the prevalence rate among the European countries. However, the prevalence rate of this disease in Isfahan, the third largest city in Iran, is 73 in 100 k people which resembles the rate in England. The mortality rate among the people with this disease in Iran is anxiously higher than the rest of the world [2]. Furthermore, numerous social and economic consequences of this disease are being imposed to the society, due to the destructive and disabling nature of this disease, particularly among the young people.

There are studies that have identified the influential factors in the formation of MS disease including immunodeficiency, genetics, viral diseases (e.g., Epstein-Barr), infectious mononucleosis, and influenza. These studies have also concluded that the MS symptoms are fatigue (75–90%), weakness (30.8%), optic neuritis symptoms (20.1%), nerve damage (19.6%), and ataxia (14.3%) [3–5].

As explained above, the most common problems in MS patients is fatigue. There are various definitions of fatigue in patients with MS, the most important and comprehensive definition is the one presented by the American MS Association in 1998: “A subjective feeling of lack of physical and mental energy to perform and complete routine and favorite activities that is recognized by the caregiver or by the patient itself” [6]. A study conducted by Papalardo and Reggio found that 80% of MS patients suffer from fatigue which effects on their daily activities and affairs, some of whom lose their jobs because of fatigue. This will thus reduce individuals’ ability to perform individual and social tasks, work, activity and maintaining a normal life [7]. It is also reported that 71% of MS patients have more than usual sick-days off work, such that 28% of patients were forced to resign due to the severe fatigue, and 75% of patients were forced to change their jobs because of this, and had no choice but to accept other jobs with considerably lower income [8]. The pharmaceutical and non-pharmacological methods may be used to alleviate this severe fatigue symptom. Among the pharmaceutical medicines, amantadine and pamulin, which have many side effects, could generate more difficulties for the MS patients [8, 9]. In addition to pharmaceutical therapies, non-pharmacological methods have also attracted attention recently among the MS patients. These treatments are based on a community-based approach which can be used to enhance the patient’s physical and mental well-being through various methods including touch therapy, hypnosis, physical exercise, aromatherapy, acupuncture, acupressure, etc. [10, 11]. Sutherland and Anderson argued that just as the exercise has a beneficial effect on the physical ability of healthy individuals, it also has potential effects to reduce the risk of physical and mental illness. However, they strongly advise the MS patients to avoid severe exercise to minimize relapse of the disease and increase the fatigue resulted from the sever exercise. The MS patients should benefit from the constructive effects of the acceptable level of physical exercise as an important part of their treatment and lifestyle. A similar recommendation that regular exercise preserves physical and mental health and is able to reduce the risk of developing chronic diseases, increase the chance of survival and improve the quality of life over the years to come was also given in [12, 13]. It should be noted that the regular physical exercise allows the different parts of the muscle to interact to each other. As a result, there would be a desired range of motion in each joint, which is essential for maximum flexibility. The flexibility attained out of exercise is important not only for physical activity but also to prevent further injury. Inactivity among the MS patients causes the joints to lose their flexibility as the connective tissues become shorter [14].

There are several preliminary studies regarding the effects of exercise on fatigue reduction in MS patients. In a recent study conducted in Iran [15], the mean fatigue score before exercise was 1.43 and after exercise 8.32. In a similar study in Ireland [16], these scores were respectively 3, and 20. The mean fatigue score before and after exercise in the Switzerland experiment [17], were 1.5 and 4.4, respectively. One of the benefits of conducted meta-analysis in this paper is that it would provide plausible responds to the assumptions discussed above. The purpose of this study is thus to determine the effect of the physical exercise on fatigue in patients with MS in Iran and the world using a comprehensive meta-analysis.

## Methods

### Method of searching articles

The Persian databases of SID, MagIran, IranMedex and IranDoc; and the international databases of Google scholar, Cochrane, Embase, ScienceDirect, Scopus, PubMed and Web of Science (ISI) were all thoroughly searched in this study with this objective of finding relevant studies related to the aim of this study. The list of relevant references used in all related articles and reports published from 1996 to 2019 in the above electronic databases were selected and manually reviewed to find other possible sources. The keywords used to search for sources were selected from the MESH Medical Topics Database. The Persian keywords used in this systematic review were exercise, fatigue, MS and multiple sclerosis; and the Latin keywords were exercise, Aerobic exercise, exercise training, physical activity, lassitude, fatigue, multiple sclerosis, sclerosis and MS.

### Selection criteria for articles

Articles with the following characteristics were selected for meta-analysis: 1) original research articles; 2) clinical trial studies; 3) full text articles and reports; 4) studies examining the relationship between exercise and fatigue in MS patients, and articles published in Persian with English abstract (native language of the researchers) and English (official language of international published articles).

### Exclusion criteria for articles

The review studies included systematic review, meta-analysis, duplicate studies; the observational studies included case-control and cohort studies (i.e., the studies at which the sample was selected from non-MS patients); as well as the studies repeated with previous data were all excluded from the meta-analysis.

### Extracting the data

The final selected articles entered into the meta-analysis process were prepared to be extracted using a pre-prepared checklist. The checklist includes article title, first author’s name, year of publication, place of study, sample size of intervention group, sample size of control group, mean sample before intervention, mean sample after intervention, standard deviation of sample before intervention, standard deviation after intervention, and probability value.

### Statistical analysis

Considering that the studied index was the effect of the exercise on fatigue in MS patients, frequency and percentage as well as the standardized mean difference index in each study were used to combine the results of different studies. The I^2^ index was used to investigate homogeneity between the studies. Due to heterogeneity in the selected studies, random effects model was used to combine these studies, and then perform meta-analysis. The *P*-value less than 0.05 was considered significant. The funnel plot and Egger test were also used to evaluate the publication bias.

## Results

**Fig. 1:**
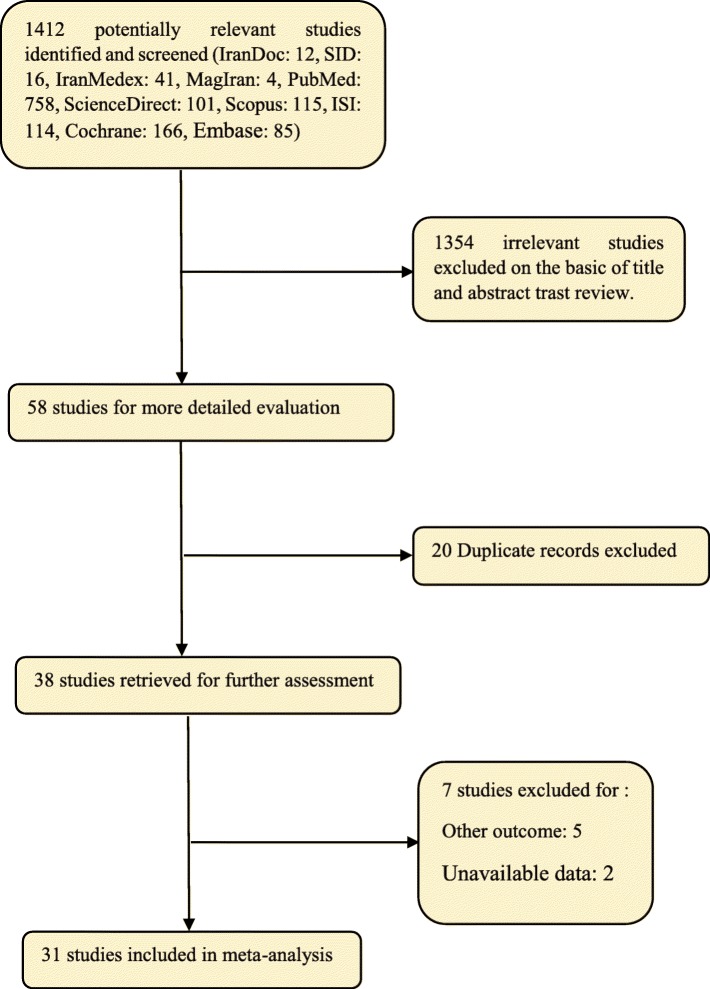
Flow diagram of study selection

In this study, all studies regarding the effect of the exercise on fatigue in patients with MS in Iran and the world without time limitation were systematically reviewed based on PRISMA checklist and PRISMA flow diagram. In the initial search, 1412 articles were identified, where eventually 31 studies, published between 1996 and September 2019, were included in the final analysis (Fig. 1).

The total number of participants selected in the meta-analysis was 1434 (720 in the control group and 714 in the intervention group). The characteristics of the studies included in the systematic review are shown in Table 1. It should be noted that all selected studies were clinical trials. Out of 31 articles selected to conduct the meta-analysis, 15 of them were published in Persian, and the rest were published in English (Table 1).

**Table 1:** Specifications of studies entered into the meta-analysis

Author, year, Reference	Place of study	clinical form of the disease (Expanded Disability Status Scale (EDSS)	type of exercise	sample size Control group	sample size intervention group	Mean ± SD of Before	Mean ± SD of After	*P*-Value
Kargarfard, 2018, [15]	Iran	Progressive <5.5	Aquatic Exercise	15	17	43.10 ± 14.60	32.80 ± 5.91	0.01
Pazokian, 2013, [18]	Iran	Progressive <5.5	Aerobic Exercise	40	40	42.95 ± 15.02	43.1 ± 14.6	0.001
Pazokian, 2012, [19]	Iran	Progressive <5.5	Aerobic Stretching‌ Exercise	40	40	51.35 ± 12.83	28.17 ± 10.32	0.001
Khodadadi, 2012, [20]	Iran	progressive 4.26 ± 1.48	Frankel exercises	15	15	4.86 ± 1.18	3.20 ± 0.94	0.0001
Asadi zaker, 2010, [21]	Iran	progressive 3.03 ± 1.63	Aerobic Stretching‌ Exercise	15	15	5.1 ± 1.1	1.80 ± 0.93	0.001
Shanazari, 2012, [22]	Iran	progressive <4.5	Aquatic Exercise	19	19	72.47 ± 5.99	40.20 ± 8.30	0.05
Ebrahimi-1, 2013, [23]	Iran	Progressive 2.6 ± 0.73	Resistance Exercise	20	20	3.98 ± 1.40	2.84 ± 1.20	0.15
Ebrahimi-2, 2013, [23]	Iran	Progressive 2.65 ± 0.91	Endurance exercise	20	20	4.39 ± 1.56	3.64 ± 1.71	0.48
Khademolsharieh, 2018, [24]	Iran	Progressive 3.4 ± 0.5	Endurance- Resistance exercise	10	10	32.91 ± 4.87	26.25 ± 7.57	0.001
Razavi, 2015, [25]	Iran	Progressive 2.04 ± 1.01	Aqua Gymnastic Exercises	24	24	4.68 ± 1.47	3.23 ± 1.51	0.017
Shaeifi, 2013, [26]	Iran	Progressive 1–5	Endurance- Resistance exercise	35	35	31.07 ± 5.17	23.40 ± 5.18	0.06
Abasi, 2016, [27]	Iran	Progressive <4.5	Rehabilitation exercises	48	48	64.23 ± 3.3	30.05 ± 3.1	0.05
Moradi, 2015, [28]	Iran	Progressive 3.57 ± 0.85	8 weeks of resistance training	15	15	3.23 ± 2.50	2.23 ± 1.21	0.029
Negaresh, 2019, [29]	Iran	Progressive 1.8 ± 0.8	short-term interval exercise	14	17	3.40 ± 0.50	3.20 ± 0.70	0.06
Ghaffari, 2008, [30]	Iran	Progressive <5.5	Aquatic Exercise	25	25	5.48 ± 0.71	2.56 ± 0.65	0.001
Eftekhari, 2008, [31]	Iran	Progressive 2.1 ± 0.4	Endurance exercise	24	24	10.80 ± 3.90	9.00 ± 4.50	0.015
Kooshiar, 2015, [32]	Iran	Progressive <6	Aerobic Exercise	20	20	41.75 ± 8.33	35.06 ± 12.20	0.001
Ghajarzadeh, 2013, [33]	Iran	Progressive 5.3 ± 2.1	Aerobic Exercise	75	75	29.70 ± 17.00	13.80 ± 14.10	0.001
Moghadas, 2017, [34]	Iran	Progressive 2.17 ± 0.92	Neurofeedback Training	10	8	5.27 ± 0.23	5.21 ± 0.21	0.104
Belochi, 2012, [35]	Iran	Progressive <5.5	Cauthorn and Coxy exercises	15	15	3.65 ± 2.54	2.23 ± 1.21	0.842
McCullagh, 2008, [16]	Ireland	Progressive 2–5	Long-term exercise	12	12	20.0 ± 2.5	3.0 ± 0.8	0.02
Mostert, 2002, [17]	Swiss	Progressive <4.5	4 weeks of aerobic exercise	24	12	5.10 ± 1.80	4.40 ± 1.90	–
Surakka-1, 2004, [36]	Finland	Progressive <6	aerobic exercise	31	30	27.30 ± 2.20	26.30 ± 2.00	–
Surakka-2, 2004, [36]	Finland	Progressive <6	strength exercise	17	17	24.80 ± 3.10	23.50 ± 2.90	–
Learmonth, 2012, [37]	England	Progressive 6.14 ± 0.36	12-week group exercise	12	20	5.70 ± 1.20	5.30 ± 1.70	0.108
Kileff, 2005, [38]	England	Progressive <6	aerobic exercise	15	15	50.29 ± 6.42	40.57 ± 8.92	0.058
Oken, 2004, [39]	USA	–	aerobic exercise	15	15	13.20 ± 4.00	12.10 ± 1.80	0.01
White, 2004, [40]	USA	Progressive 1–5	resistance training	5	5	32.00 ± 18.00	25.00 ± 8.00	0.04
Vore, 2011, [41]	USA	Progressive 2–5	10-week group exercise	11	11	26.90 ± 5.85	21.82 ± 8.88	–
Petajan, 1996, [42]	USA	Progressive 3.8 ± 0.3	aerobic exercise	25	21	48.70 ± 2.00	44.40 ± 1.8	0.05
Mathiowetz, 2001, [43]	USA	–	13-week group exercise	54	54	22.00 ± 7.70	17.30 ± 8.20	–

According to the available data, standardized mean difference indices and relative risk were used to finalize the effects of the studies. In the studies that the standard deviation of ± of mean were reported, that standardized mean difference index was used in the meta-analysis. The results of the meta-analysis showed that heterogeneity between the studies was obtained to be (*I*^2^ = 99) before the intervention and (*I*^2^ = 99) after the intervention. Therefore, the random effects model was used to combine these studies to derive the final outcome.

**Fig. 2:**
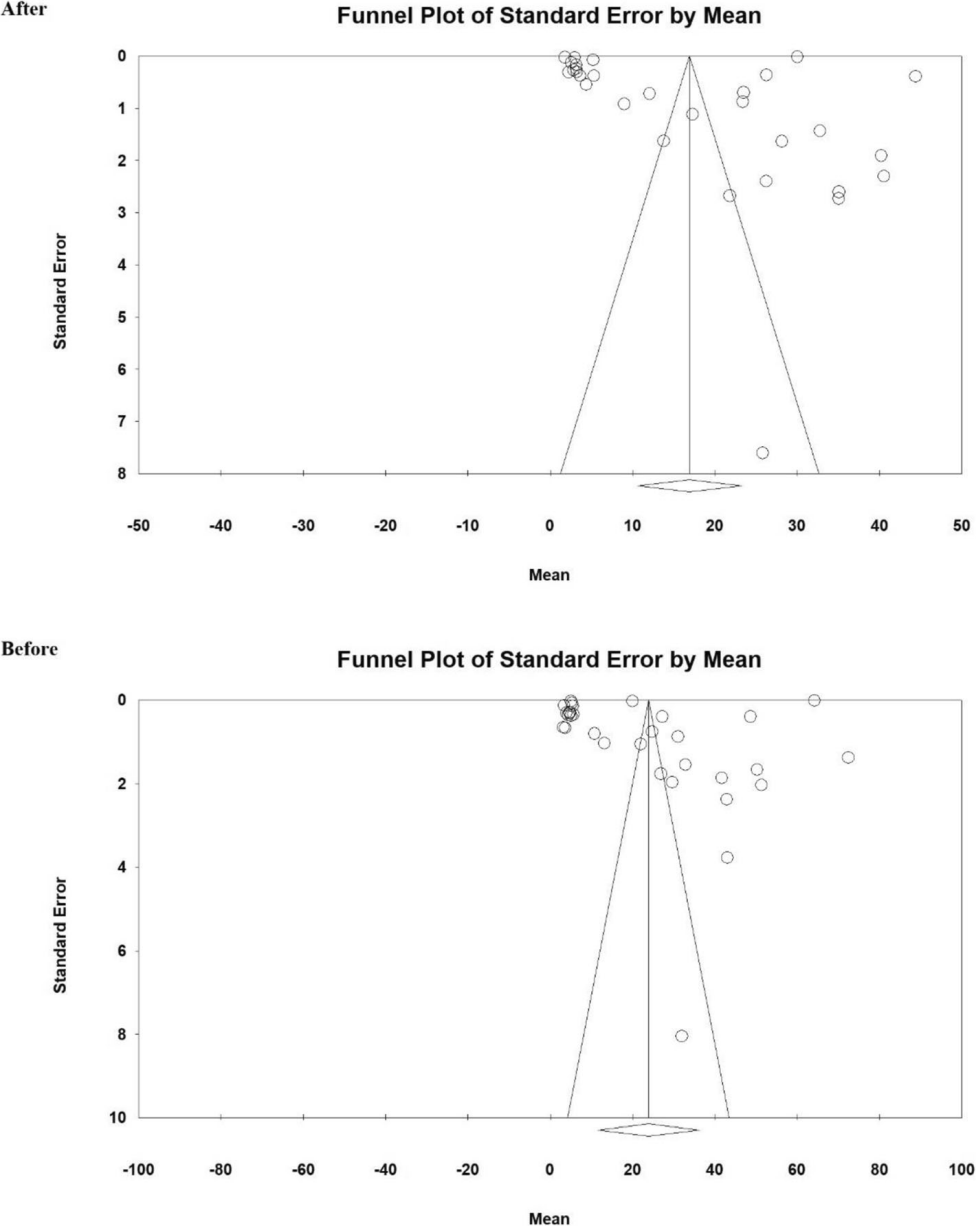
Funnel plot from studies included in the meta-analysis using standardized mean difference index before and after intervention

The Egger test was used to investigate the presence of bias in the studies. According to the Egger test results, there was no publication bias in the studies before (*P* = 0.177), and after the intervention (*P* = 0.279), (Fig. 2).

**Fig. 3:**
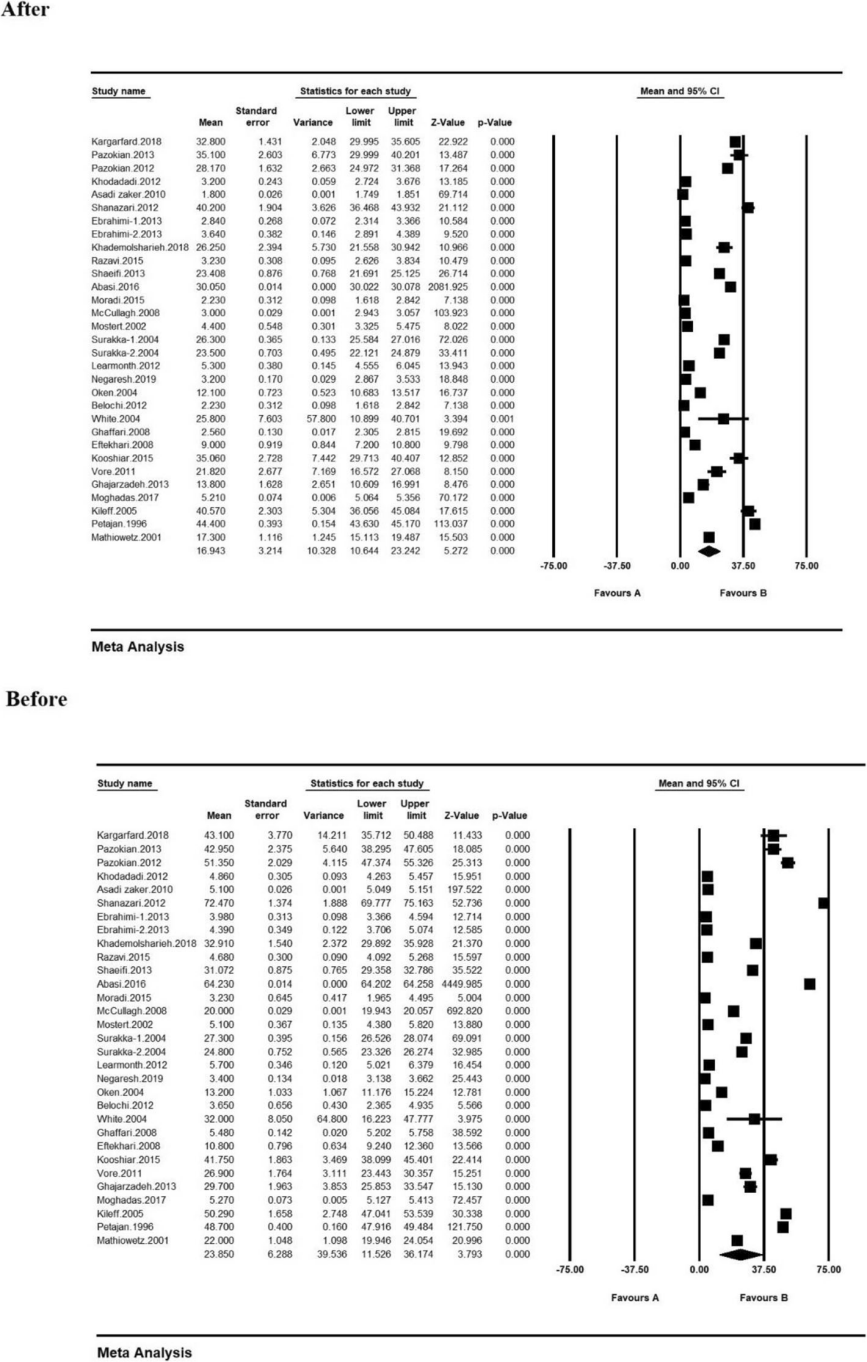
Forest plot of studies included in the meta-analysis using standardized mean difference index before and after the intervention

Based on the results derived from the meta-analysis, the standardized mean difference between the intervention groups before and after the intervention were estimated to be 23.8 ± 6.2 and 16.9 ± 3.2, respectively. This would suggest and confirm that the physical exercise will reduce fatigue in the patients with MS. In the forest plot (Fig. 3), the standardized mean difference index and its 95% confidence interval in each study, as well as the final estimation of the index from the combination of studies are all illustrated. In this figure, the weight of each study in the final combined value exhibits that the size of each square is appropriate to the weight that the study had in the meta-analysis. The horizontal line of each square also shows a 95% confidence interval (Fig. 3).

**Fig. 4:**
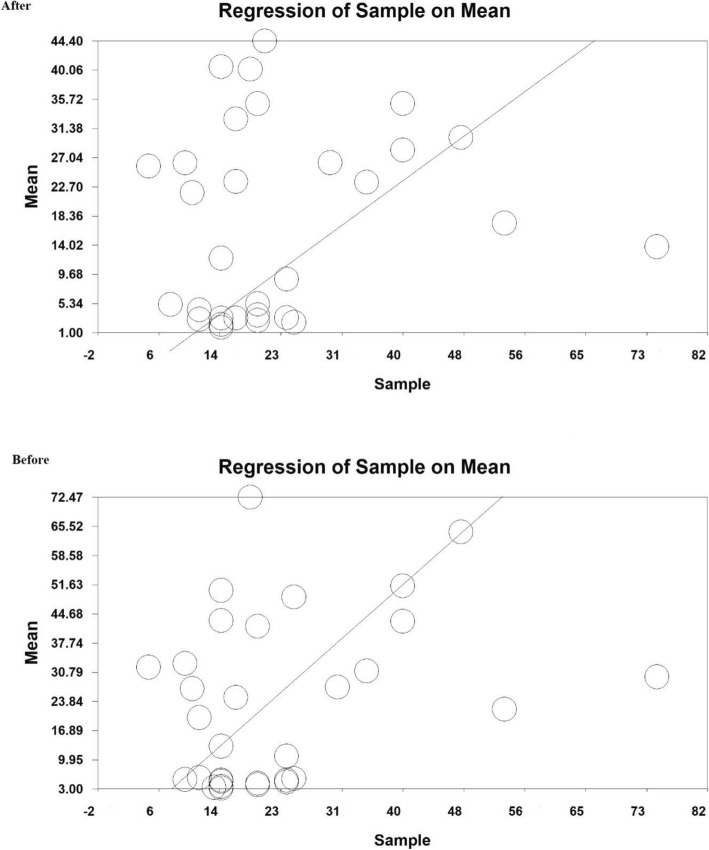
Meta-regression of the relationship between sample size and studies included in meta-analysis using standardized mean difference index before and after the intervention

**Fig. 5:**
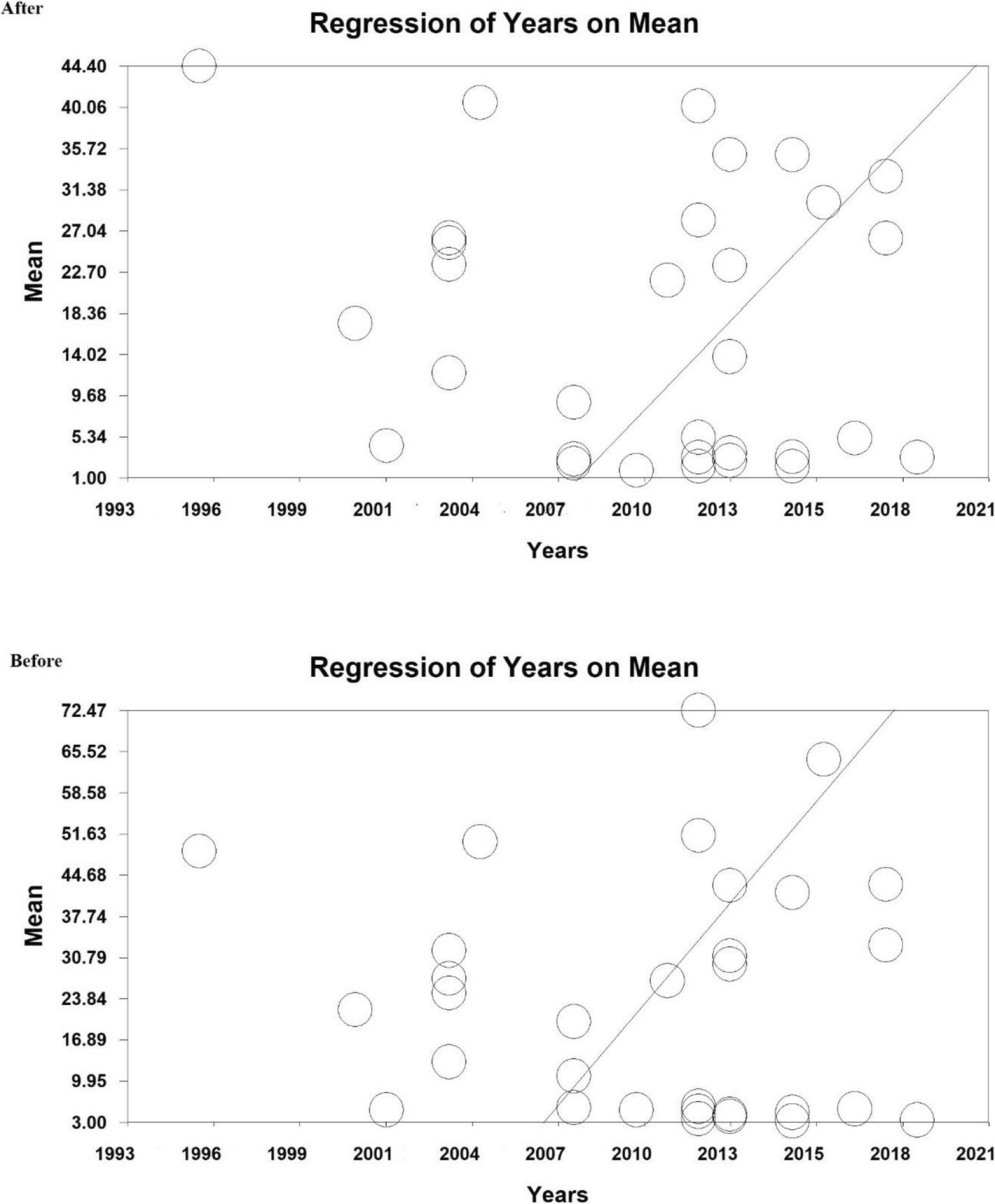
Meta-regression of the relationship between the study year and the studies included in the meta-analysis using the standardized mean difference index before and after the intervention

The meta-regression based on pre- and post-intervention, the year of doing studies (*P* = 0.000) and sample size (*P* = 0.000) with standardized mean difference before and after the intervention were investigated. As a result, a significant difference was observed between standardized mean difference before and after the intervention. The fitted models suggest that with the increase of the year of research and sample size, the standardized mean would also increase before and after the intervention (Figs. 4 and 5).

## Discussion

The purpose of this study was to determine the effect of exercise on fatigue in the patients with MS in Iran and the world using a comprehensive meta-analysis.

Fatigue is a common and annoying symptom in the MS patients and various factors such as lack of physical activity, muscle weakness and neurological problems are involved in this complication. The decreased endurance and muscle resistance would usually lead to the early fatigue and consequently decrease the level of patients’ activity level [44]. In general, the cause of fatigue in the MS patients is unclear, and has been reported in various sources as immune mechanisms, neuromuscular, thermal sensitivity, respiratory muscle weakness, and increased cost of respiratory muscles [45, 46].

The results of this study show that the severity of fatigue is higher than before the test and this indicates the importance of fatigue level and duration, and its effect on all aspects of life of these patients. A special attention must be paid to the young people as the efficient forces of society and the largest age group having this disease. Fatigue can even have devastating effects, and these patients may become an additional burden to the society. The more sedentary a patient is, the less energy he or she will have to work. This decrease in physical activity will result in reduction of muscle mass and further decrease functions [18].

The results of the present study showed that there is a significant difference between the mean scores of pre-test and post-test of fatigue severity in MS patients in the intervention group. The patients in the intervention group achieved a score of 16.9 in the fatigue severity score as a result of exercise.

The possible mechanism of fatigue reduction in the present study may be due to different and beneficial functions of exercise in the various aspects of these patients’ lives. However, the previous studies have suggested that increased skeletal muscle activity during exercise could be due to increased blood flow to the muscles. This would also increase the volume of left ventricle and consequently cardiac output during physical activity, particularly increase heart rate. On the other hand, as arterioles open in skeletal muscles, blood and oxygen transportation to muscle tissue will be increased, which eliminates the need for extra oxygen during physical activity because of increasing respiratory rate, vital lung capacity, and alveolar ventilation [28]. In fact, the early benefits of regular exercise of these patients include: increased cardio-respiratory fitness, increased muscle strength and endurance, reduced body fatigue, improved morale, and increased ability to perform daily tasks with greater force. This is also significantly effective in controlling MS symptoms and health improvement [47]. Since these patients frequently experience weakness (particularly, in the lower extremities) and fatigue; as a result of doing the exercises, they may be able to overcome the weakness in their lower extremities, which often results in fatigue and impaired walking. It will thus significantly reduce the severity of fatigue after exercise in MS patients [48–51].

In this study, we find that exercise significantly reduces fatigue in Iranian MS patients compared to other countries. Because fatigue is a mental phenomenon and difficult to measure, and influenced by many factors, such as emotional changes and other disturbing symptoms associated with MS. Therefore, this could be the possible cause of different results reported in Iranian and foreign articles. Another plausible reason could be due to younger population of the country, which results in the increased number of younger patients eventually making up a larger percentage of the patients in the intervention group.

Since chronic diseases, including MS, affect economic, financial, social, and emotional aspects of the individual, family, and community, the nurses can play an imperative role in the rehabilitation of patients with physical and mental inabilities. They are able to help patients to improve their ability to perform their daily activities and to help them eliminate social, psychological and economic burdens. A nurse could guide the patient and family in the right direction by helping them determining the ways to control their complications. Among the many types of exercise, aerobic exercises that widely consume oxygen, cause significant metabolic changes such as improving metabolism; decreasing levels of epinephrine, norepinephrine, cholesterol and triglycerides; boosting the immune system; and, improving endorphin secretion, mood and mental status. The important thing is that the demethylation process does not change during these activities. In other words, when the musculoskeletal system is inactive, the duration of oxidation reduces, and this is an important factor in the incidence of fatigue and sluggishness of daily activities. By doing exercise, the oxidation capacity of the muscles is increased, thereby the aerobic biochemical system is stimulated to make the adaption and leads to increase the amount of oxygen intake in the body. Some diseases inhibit oxygen in each of the above stages and decrease functional capacity, but aerobic exercises are able to make physiological adaption to the efficiency of the aerobic energy system, enhance one’s functional ability, even improve disease progression and improve functional capacity. The other benefits of regular exercise for these patients are increased strength, improved body status, reduced fatigue, improved mood, increased self-esteem, and a sense of well-being. Furthermore, performing the physical exercise improves one’s independence and not only improves patients’ quality of life, but also affects patients’ balance through coordinating upper and lower extremities, and prevents cardiovascular diseases, diabetes, and so on [52]. Therefore, it is recommended that practitioners use regular exercise as a complementary treatment along with medications to help patients with MS.

## Conclusion

The results of this study show that exercise significantly reduces fatigue in patients with MS. Therefore, a regular exercise program can be part of a rehabilitation program for patients with MS.

## Data Availability

Datasets are available through the corresponding author upon reasonable request.

## Abbreviations

MS: Multiple Sclerosis
PRISMA: Preferred Reporting Items for Systematic Reviews and Meta-Analysis
